# The longitudinal association between chronic disease resource utilization and self-management behaviors in newly diagnosed type 2 diabetes patients: The mediating role of health literacy

**DOI:** 10.1097/MD.0000000000050291

**Published:** 2026-08-21

**Authors:** Ming Fan, Weipeng Qian

**Affiliations:** a Department of Endocrinology, The Sixth People’s Hospital of Nantong, Nantong, Jiangsu, China.

**Keywords:** chronic disease, type 2 diabetes mellitus, health literacy, longitudinal studies, self-management, social support

## Abstract

The aim of this study is to investigate the dynamic interaction among chronic disease resource utilization, health literacy, and self-management behaviors in individuals newly diagnosed with type 2 diabetes mellitus. This study was conducted from November 2022 to August 2024 using a longitudinal design. Participants were recruited via convenience sampling from the Department of Endocrinology, Nantong Sixth People’s Hospital in Jiangsu Province. Eligible patients were those newly diagnosed with T2DM within 1 month. A total of 354 patients completed all 3 waves of the survey. Core variables, including chronic disease resource utilization, diabetes health literacy, and diabetes self-management behavior, were assessed at 3-time points: baseline (T1, approximately 7 days after enrollment), 3 months (T2), and 6 months (T3), using the Chronic Disease Resource Questionnaire, the Diabetes Health Literacy Scale, and the Diabetes Self-Management Behavior Scale, respectively. After controlling for covariates such as age, gender, and residential area, cross-lagged panel modeling and the Bootstrap method were employed to analyze longitudinal predictive relationships and mediation effects among the variables. Health literacy and self-management behavior showed reciprocal positive predictions across T1→T2 (*β* = 0.32, *P* < .01; *β* = 0.23, *P* = .016) and T2→T3 (*β* = 0.28, *P* = .003; *β* = 0.31, *P* < .001). Resource utilization significantly predicted improved health literacy at T1→T2 (*β* = 0.24, *P* < .01), but not at T2→T3 (*β* = 0.11, *P* = .108). During T1→T2, resource utilization indirectly enhanced self-management via health literacy (mediation effect *β* = 0.067, *P* = .008), accounting for 30.9% of the effect. A bidirectional promotive relationship exists between health literacy and self-management. The positive effect of resource utilization is most effective within the first 3 months post-diagnosis. During the initial phase (0–3 months post-diagnosis), efforts should focus on strengthening the integration and accessibility of resources such as community healthcare and digital health tools, aiming to rapidly improve patients’ health literacy. After 3 months, the intervention emphasis should shift towards promoting the autonomous reinforcement and maintenance of self-management capabilities through methods such as behavioral feedback and goal setting, so as to achieve precision management.

## 1. Introduction

Diabetes has become a major global public health challenge. According to the International Diabetes Federation, the number of patients worldwide is expected to exceed 600 million by 2040. Newly diagnosed type 2 diabetes mellitus (T2DM) patients face a higher risk of self-management failure due to insufficient disease awareness and lack of coping experience.^[1]^ Studies have shown that at the time of initial diagnosis, patients still retain some pancreatic β-cell function, with residual insulin secretion reaching 20 to 50% of the normal level.^[2]^ Through intensive intervention (such as low-carb diets and short-term insulin therapy), partial reversal of β-cell function is possible. However, in China, the rate of achieving blood glucose control targets within 1 year for newly diagnosed T2DM patients is only 29.5%,^[3]^ significantly lower than that in developed countries. The core contradictions lie in 2 aspects: first, there is a structural deficiency in health literacy,^[4]^ with patients of lower income and education levels having limited health decision-making abilities, directly affecting their understanding of information and compliance with actions; second, the utilization of chronic disease resources is inefficient,^[5,6]^ with insufficient accessibility of traditional community medical resources and inadequate integration of digital health tools into the management loop.

The synergy between health literacy and chronic disease resource utilization is the key pathway to optimizing diabetes management. Systematic reviews have shown that insufficient health literacy can increase the risk of self-management failure in T2DM patients by 1.8 times,^[7]^ while resource utilization behaviors, such as community medical support and Internet platform management, can significantly improve glycated hemoglobin A1c (HbA1c) and self-efficacy by enhancing patient activation.^[8,9]^ Longitudinal studies further revealed that health literacy has a 2-way empowerment effect, and the health literacy of patients and family caregivers positively predicts self-management behavior. Six months after diagnosis is the critical intervention period for the formation of self-management behavior, and resource integration during this period can maximize the transformation efficiency of health literacy.^[10]^ Although the causal relationship between “health literacy → self-management” has been established,^[11]^ there are still 3 bottlenecks in the current research.

Currently, there are several issues concerning the utilization of chronic disease resources, health literacy, and self-management behaviors among individuals newly diagnosed with T2DM. Firstly, the dynamic relationship between these factors is not well defined. Most studies, including that of Bains et al,^[12]^ utilize a cross-sectional design, which fails to discern the causal sequence between health literacy and self-management. Secondly, there is a deficiency in the mediation mechanism. The process by which chronic disease resource utilization influences behavior through health literacy lacks verification through a longitudinal mediation model.^[13]^

This study directly addresses the aforementioned gaps, which constitute its primary innovation and necessity. First, innovation in methodology and perspective: this study moves beyond the static, cross-sectional viewpoint by employing a 3-wave longitudinal design integrated with cross-lagged panel modeling. This innovative approach allows for a rigorous analysis of the dynamic interactions and temporal causal sequences among chronic disease resource utilization, health literacy, and self-management behavior in patients newly diagnosed with T2DM. Few studies have applied such temporal analysis methods to deeply examine the evolving relationships among these 3 core constructs during the critical 6-month post-diagnosis window. Second, innovation in theoretical mechanism validation: the study’s innovation lies in explicitly testing a longitudinal mediation model. It aims to empirically examine whether health literacy serves as a key psychological mechanism that translates the utilization of external resources (i.e., chronic disease resources) into specific self-management actions. This shifts the research focus from merely associating resources with outcomes to understanding the process through which resources exert their effects, that is, the mediating pathway. Third, necessity for evidence-based practice: the findings of this study are crucial for developing precise, phased intervention programs. By identifying when resource utilization has the strongest impact on health literacy and when the reciprocal relationship between health literacy and self-management behavior is most pronounced, this research will provide much-needed evidence-based support for constructing a “healthcare provider–patient–family” triadic precision support model. This will guide healthcare systems and clinical practitioners in strategically deploying resources at optimal time points to maximize patient empowerment and long-term management outcomes.

Therefore, this study aims to utilize longitudinal design and cross-lagged analysis to elucidate the dynamic interplay and mediating mechanisms among chronic disease resource utilization, health literacy, and self-management behavior in patients newly diagnosed with T2DM. Ultimately, it seeks to establish a theoretical foundation for developing precise, family-empowered intervention programs tailored to the patient’s post-diagnosis disease trajectory.

## 2. Subjects and methods

### 2.1. Subjects

This study is a secondary analysis of a subset of data from our previously published prospective cohort study.^[14]^ From November 2022 to August 2024, a convenience sampling method was employed to select newly diagnosed T2DM patients who were treated in the outpatient and inpatient departments of the Endocrinology Department at the Sixth People’s Hospital of Nantong City, Jiangsu Province, as the subjects of the research. Inclusion criteria were as follows: patients met the diagnostic criteria for newly diagnosed T2DM as outlined in the Chinese Guidelines for the Prevention and Treatment of Type 2 Diabetes (2020 edition),^[15]^ with a diagnosis time of no more than 1 month; age of 18 years or older; a complete family support system, with at least 1 adult family member, such as a spouse, children, or parents, able to provide care support; basic reading and writing skills, enabling them to independently complete the questionnaire. Exclusion criteria: severe complications, such as diabetic ketoacidosis, severe retinopathy, diabetic nephropathy with estimated glomerular filtration rate < 30 mL/min/1.73 m^2^, or acute cardiovascular and cerebrovascular events within the past 6 months; pregnant or lactating women; patients expected to be unable to complete 3 follow-up visits during the study period, such as those on long-term business trips or living in remote areas; those with severe mental illness (such as major depression or schizophrenia) or cognitive impairment (Mini-Mental State Examination score ≤ 23). Based on the data requirements and statistical power requirements of the longitudinal cross-lag model, this study determined the sample size through the following 2 criteria. First, model complexity criteria: according to the sample size criterion of structural equation modeling (SEM),^[16]^ the sample size should be more than 10 times the number of free parameters in the model. The cross-lag model involved autoregressional and cross-lag paths of 3 variables (chronic disease resource utilization, health literacy, and self-management behavior) at 3-time points. A total of 27 core path coefficients needed to be estimated, so the minimum sample size required was 27 × 10 = 270 cases. Second, compensation standard for loss to follow-up rate: referring to the average loss rate of 25% in the China Diabetes Cohort Study,^[17]^ the baseline sample size was adjusted using the formula: N baseline = N end/(1 − loss to follow-up rate) = 270/(1−0.25) = 360. Consequently, 360 newly diagnosed T2DM patients were included at baseline to ensure that the effective sample at T3 was ≥ 270. The study was approved by the Ethics Committee of the Sixth People’s Hospital of Nantong (approval number: NTLYLL2022073), and all patients signed informed consent.

### 2.2. Survey tools

#### 2.2.1. General information questionnaire

A self-developed baseline information collection form was utilized in this study, encompassing 3 dimensions of indicators. Demographic characteristics included age, gender, marital status, and type of permanent residence; socioeconomic characteristics comprised education level, family per capita monthly income, and employment status; clinical outcomes focused on the latest HbA1c level.

#### 2.2.2. Chronic disease resources questionnaire

The scale was developed by the Glasgow team^[18]^ in 2005 and subsequently localized and revised by the Zhong Huiqin research group.^[19]^ It comprises 6 functional domains: medical professional support, social network, self-regulation strategy, community environmental resources, information dissemination system, and organizational support system. A total of 19 items are measured. A 5-point Likert scale is employed to assess the frequency of resource utilization, with options ranging from 1 to 5, corresponding to “never used” to “fully used.” The theoretical total score spans from 19 to 95 points. The scale demonstrates good reliability and validity among diabetic patients, with a Cronbach *α* coefficient of 0.836 in this study.

#### 2.2.3. Diabetes health literacy scale

The diabetes health literacy-specific scale for cross-cultural adaptation was developed by Professor Ishikawa^[20]^ and completed by Zhao Xiaoyan^[21]^ in 2021. The scale constructed 3 core dimensions of functional health literacy, communication health literacy, and critical health literacy, and contained 14 specific evaluation items. A 4-point Likert scale was used (1 = can’*t* do it at all, 4 = can do it at all), and the functional dimension items were scored in reverse (the score conversion was included in the total score). The total score on the scale ranges from 14 to 56, with higher scores indicating better levels of health literacy. The Chinese version of the validation study showed that the Cronbach *α* of the total scale was 0.868, and the *α* coefficients of the 3 dimensions were 0.839 (communication), 0.812 (critical), and 0.765 (functional), which exceeded the psychometric acceptability threshold (*α* > 0.7).

#### 2.2.4. Diabetes self-management scale

This scale was constructed by Toobert et al^[22]^ in 2000, and Sun^[23]^ completed cross-cultural adaptation and verification in 2011. The core framework contained 6 dimensions, namely diet, exercise, blood glucose monitoring, foot care, and medication compliance, with a total of 11 items. Using the behavior frequency assessment method in the past 7 days (0 = not performed, 7 = performed every day), the maximum score of each item was 7, and the total score ranged from 0 to 77. The higher the total score, the better the self-management behavior compliance. The Cronbach *α* coefficient of the questionnaire in this study was 0.871, and the reliability reached the standard (0.7).

### 2.3. Data collection methods

This study employed a convenience sampling method. Potential participants were recruited from the outpatient and inpatient departments of the Department of Endocrinology at the Sixth People’s Hospital of Nantong City, Jiangsu Province. The recruitment period spanned from November 2022 to August 2024, and the target population consisted of patients newly diagnosed with T2DM.

The specific participant selection and recruitment process was as follows: patients who met the predefined inclusion and exclusion criteria were invited to participate in the study after the research team explained the study’s objectives and procedures. Ultimately, a total of 385 eligible patients who provided informed consent were enrolled and completed the T1 baseline data collection, forming the initial study cohort.

Based on the evidence of the window period for diabetes management behavior, the effect of behavioral intervention became apparent within 3 months, and the requirement for continuous observation for chronic disease management is for at least 6 months.^[10]^ Consequently, this study employed a 3-point longitudinal tracking design. Assessments were conducted 7 days post-enrollment (the standardized evaluation point following health education), at 3 months post-enrollment at T2 (the critical period for behavior change), and at 6 months post-enrollment at T3 (the effect consolidation evaluation point). The standardized instructions and mixed investigation mode were executed by 5 clinical nurses who underwent unified training to enhance compliance. Demographic characteristics and baseline scale data (Chronic Disease Resource Utilization Scale, Diabetes Health Literacy Scale, Self-Management Behavior Scale) were collected at T1. At T2 and T3, the scales were readministered through on-site reexamination (72.3%), video interview (18.9%), or telephone follow-up (8.8%) after WeChat/telephone appointment. A total of 385 newly diagnosed T2DM patients were included in the study, with 354 patients (91.95%) completing all 3 surveys, and 31 patients (8.05%) lost to follow-up. The primary reasons for loss to follow-up were refusal to continue participation (n = 28) and loss of contact (n = 3). There were no significant differences in gender distribution (*χ*^2^ = 0.30, *P* = .584), baseline chronic disease resource utilization level (*t* = 0.85, *P* = .396), health literacy (*t* = 0.92, *P* = .358), and self-management behavior (*t* = 0.75, *P* = .454) between the follow-up loss group and the completion group (*P* > .05). This indicates that the mechanism of missing data is consistent with the assumption of randomness and does not affect the internal validity of the longitudinal analysis. Quality control measures included: investigators underwent consistency training (scale interpretation Kappa value ≥ 0.87); 10% of the samples were randomly selected for secondary verification, and the completeness rate of item filling was 99.1%.

### 2.4. Statistical methods

A prospective longitudinal design was employed to analyze the dynamic relationship among chronic disease resource utilization, health literacy, and self-management behavior using a multi-stage mixed modeling strategy. The Friedman test was utilized to analyze the T1/T2/T3 scores of family support, health literacy, and self-management behavior. The Wilcoxon signed-rank test was applied for pairwise comparisons, and the Bonferroni correction was implemented to control for errors in multiple comparisons. A cross-lagged path model of chronic disease resource utilization → health literacy → self-management behavior was constructed using SEM to assess the causal relationship between variables. The model’s fitness was evaluated based on the following indicators: chi-square to degree of freedom ratio (*χ*^2^/df < 3), comparative fit index (CFI > 0.90), root mean square error of approximation (RMSEA < 0.08), and standardized root mean square residual (SRMR < 0.08). The significance of path coefficients was tested using the bias-corrected bootstrap method. Data analysis was conducted using SPSS 24.0 (for descriptive statistics, correlation analysis, and Friedman test) and AMOS 21.0 (for SEM modeling). R 4.3.1 (with the lavaan package; The R Foundation for Statistical Computing) was utilized for graphics and effect size calculations.

## 3. Results

### 3.1. General information of newly diagnosed T2DM patients

Among the 354 patients, ages ranged from 27 to 69 years old [median (interquartile range): 48 (37, 55)], comprising 218 males and 136 females. For detailed information, please refer to Table 1.

**Table 1:** General information survey of newly diagnosed T2DM patients (n = 354).

Items	Categories	Number	Percentage (%)
Age (years)	< 45	74	20.90
	45*–*60	195	55.09
	> 60	85	24.01
Gender	Male	218	61.58
	Female	136	38.42
Education level	Primary school or lower	22	6.21·
	Junior/High school	212	59.89
	College or above	120	33.90
Residence	Urban	228	64.41
	Rural	126	35.59
Marital status	Married	321	90.68
	Unmarried/divorced/widowed	33	9.32
Monthly household income (yuan)	< 1500	67	18.93
	1500*–*3000	164	46.33
	> 3000	123	34.74
Baseline HbA1c (%)	7*–*9	199	56.21
	> 9	155	43.79

HbA1c = glycated haemoglobin, n = number of patients, T2DM = type 2 diabetes mellitus.

### 3.2. Scores of chronic disease resource utilization, health literacy, and self-management of newly diagnosed T2DM patients

**Figure 1.**
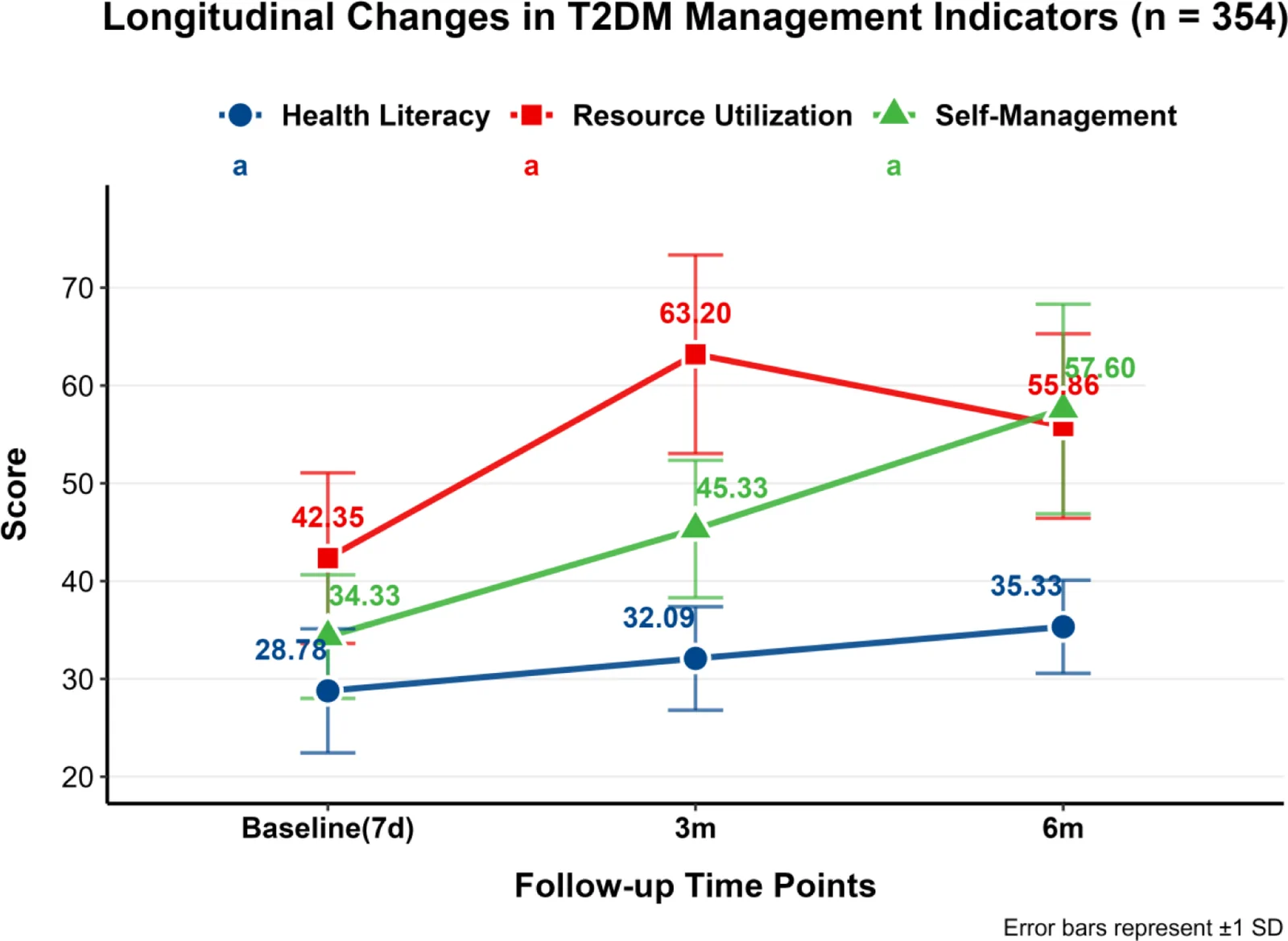
Longitudinal trends in health literacy, resource utilization, and self-management. n = number of patients, T2DM = type 2 diabetes mellitus.

Based on the 3-time-point repeated measures analysis of variance (which met the sphericity assumption and utilized the Greenhouse–Geisser correction with *ε* > 0.85), the main effects of time on chronic disease resource utilization, health literacy, and self-management ability were all significant (*F* ≥ 27.709, *P* < .001). After applying the Bonferroni–Holm correction, resource utilization exhibited a trend of initial increase followed by a decrease (3 months > 6 months > baseline), whereas health literacy and self-management ability demonstrated consistent improvement (baseline < 3 months < 6 months). For further details, refer to Table 2 and Figure 1.

**Table 2:** Longitudinal comparison of chronic disease resource utilization, health literacy, and self-management scores in newly diagnosed T2DM patients (n = 354).

Time point	Chronic disease resource utilization (score)	Health literacy (score)	Self-management ability (score)
T1: 7 days after diagnosis	42.35 ± 8.72	28.78 ± 6.34	34.33 ± 6.32
T2: 3 months after diagnosis	63.20 ± 10.15	32.09 ± 5.29	45.33 ± 7.02
T3: 6 months after diagnosis	55.86 ± 9.43	35.33 ± 4.76	57.60 ± 10.72
*H*-statistic	38.217	27.709	45.717
*P*	< .001	< .001	< .001
Post hoc comparisons	T2 > T3 > T1	T1 < T2 < T3	T1 < T2 < T3

Data presented as mean ± standard deviation. Kruskal–Wallis H-test used for nonparametric comparisons.

n = number of patients, T2DM = type 2 diabetes mellitus.

### 3.3. Correlation of chronic disease resource utilization, health literacy, and self-management ability in newly diagnosed T2DM patients

The statistical description of each variable and the outcomes of the correlation analysis are presented in Table 3. At time 1 (T1), chronic disease resource utilization exhibited a positive correlation with health literacy (*r* = 0.31) and self-management ability (*r* = 0.28). Additionally, health literacy was significantly correlated with self-management ability (*r* = 0.24). At time 2 (T2) and time 3 (T3), there continued to be significant positive correlations among the 3 variables (*r* = 0.18–0.61, all *P* < .01), fulfilling the criteria for cross-lag analysis.

**Table 3:** Correlation matrix of chronic disease resource utilization, health literacy, and self-management ability (n = 354).

Variable	1	2	3	4	5	6	7	8	9
T1 Resource utilization	1								
T1 Health literacy	0.31	1							
T1 Self-management	0.28	0.24	1						
T2 Resource utilization	0.58	0.33	0.22	1					
T2 Health literacy	0.35	0.51	0.18	0.29	1				
T2 Self-management	0.25	0.36	0.53	0.34	0.38	1			
T3 Resource utilization	0.49	0.27	0.19	0.62	0.30	0.26	1		
T3 Health literacy	0.30	0.56	0.25	0.33	0.54	0.32	0.28	1	
T3 Self-management	0.27	0.29	0.49	0.31	0.35	0.61	0.34	0.33	1

T1, T2, T3 represents the time points at diagnosis, 3 months, and 6 months post-diagnosis.

n = number of patients.

### 3.4. Cross-lag analysis of chronic disease resource utilization, health literacy, and self-management ability

**Figure 2.**
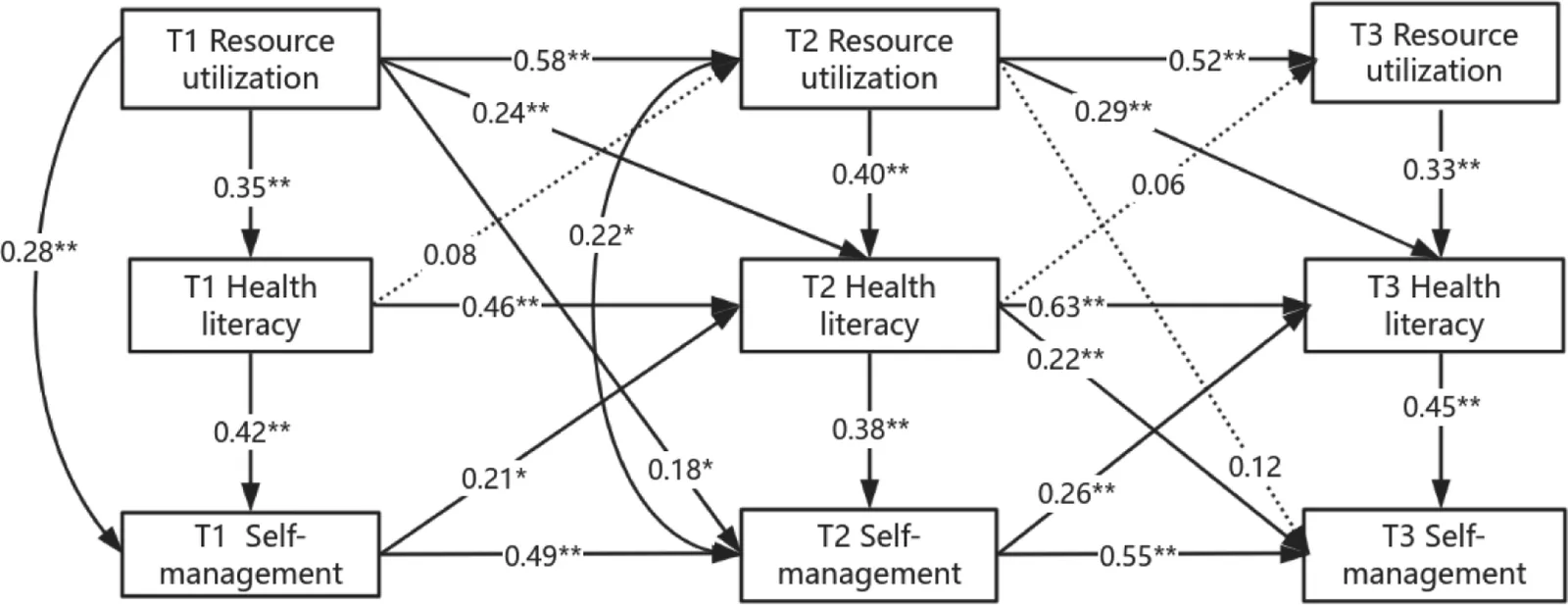
Cross-lagged analysis of resource utilization, health literacy and self-management ability.

The longitudinal cross-lagged model, based on AMOS (Fig. 2), exhibited a good fit: *χ*^2^ = 92.36, df = 31, *χ*^2^/df = 4.105 (*P* < .001), RMSEA = 0.074 (90% CI: 0.054–0.090), SRMR = 0.055, CFI = 0.96, GFI = 0.92, TLI = 0.93. After controlling for gender, age, and place of residence, the critical path effects were as follows. First, the predictive effect of resource utilization on health literacy: chronic disease resource utilization at T1 significantly and positively predicted health literacy at T2 (*β* = 0.24, *P* < .01); chronic disease resource utilization at T2 significantly and positively predicted health literacy at T3 (*β* = 0.29, *P* < .01). Second, the inverse relationship between health literacy and resource utilization: health literacy at T1 could not predict resource utilization at T2 (*β* = 0.08, *P* > .05); health literacy at T2 could not predict resource utilization at T3 (*β* = 0.06, *P* > .05). Third, the interaction between self-management ability and health literacy: self-management ability at T1 positively predicted health literacy at T2 (*β* = 0.21, *P* < .05); self-management ability at T2 positively predicted health literacy at T3 (*β* = 0.26, *P* < .01). Fourth, the dynamic correlation between resource utilization and self-management ability: resource utilization at T1 positively predicted self-management ability at T2 (*β* = 0.18, *P* < .05); T2 resource utilization did not significantly predict T3 self-management ability (*β* = 0.12, *P* > .05).

### 3.5. The utilization of resources for chronic diseases among newly diagnosed T2DM patients can longitudinally predict their self-management abilities through the mediating effect of health literacy

Nonparametric bootstrap confidence intervals were calculated for 2000 repeated samples, and health literacy played a partial mediating role between resource utilization and self-management ability, accounting for 30.9% of the indirect effect. This indicates that resource utilization not only directly promoted self-management (*β* = 0.15) but also indirectly strengthened behaviors by improving health literacy (*β* = 0.067). The fitting index was good, with *χ*^2^/df = 3.12, RMSEA = 0.073, CFI = 0.94, TLI = 0.91, and SRMR = 0.06. See Figure 2 and Table 4 for details.

**Table 4:** Longitudinal mediation analysis: health literacy as a mediator between resource utilization and self-management ability (n = 354).

Effect type	Effect size	Proportion (%)	SE	*P*	95% Cl
Direct effect					
T1 Resource utilization → T3 Self-management	0.15	69.1%	0.07	.032	0.03*–*0.27
Indirect effect					
T1 Resource utilization → T2 Health literacy → T3 Self-management	0.067	30.9%	0.02	.008	0.02*–*0.12
Total effect	0.217	100%	0.05	< .001	0.13*–*0.31

Effect sizes are standardized coefficients (*β*); Proportion = (Direct/Indirect effect ÷ Total effect) × 100%; Bootstrap confidence intervals based on 2000 resamples.

Mediation ratio = Indirect effect/ Total effect = 30.9%.

CI = confidence interval, n = number of patients, SE = standard error.

## 4. Discussion

To examine the longitudinal relationship among chronic disease resource utilization, health literacy, and self-management ability in newly diagnosed T2DM patients, a questionnaire survey was conducted 3 times over a 6-month period. The correlation between chronic disease resource utilization, health literacy, and self-management ability in newly diagnosed T2DM patients was preliminarily confirmed, and the longitudinal mediating effect of health literacy in this patient group was identified.

The utilization level of chronic disease resources in newly diagnosed T2DM patients increased first and then decreased. This study revealed that the utilization of resources for chronic diseases among newly diagnosed T2DM patients significantly increased within the first 3 months post-diagnosis (T1→T2). However, a downward trend was observed in the subsequent 3 months (T2→T3). The average scores fluctuated between 42 and 63 points across the 3-time points, aligning with the findings of Qian Yan^[24]^ and Zuo Manfang.^[25]^ This dynamic may reflect the varying needs of patients at different stages of the disease. In the initial phase post-diagnosis, patients exhibit a strong motivation to obtain resources due to the novelty of the disease^[26]^ and actively seek medical support. Common behaviors include follow-up consultations, participation in community diabetes management courses, and the use of digital health tools such as blood glucose management apps. Resource utilization at this stage demonstrates a rapid increase. During the disease adaptation period,^[27,28]^ as patients gradually acquire basic management skills, their reliance on certain resources, like frequent hospital follow-ups, diminishes. Nonetheless, the limited accessibility of primary medical resources, such as community health stations, may lead to an overall decrease in utilization rates. This observation is consistent with the “window period of resource utilization” theory proposed by Glasgow et al,^[29]^ which suggests that patients in the early stages of chronic diseases preferentially utilize available resources due to high information demand. However, without a sustained resource matching mechanism, the utilization rate may decline due to shifting demands or inadequate supply. This is in line with the phenomenon observed by Ranjbaran et al^[30]^ in their research on the health literacy and health promotion behaviors of urban adult populations, that is, the acquisition of basic resources can effectively enhance behavioral compliance in the early stage of intervention, but if the supply of resources fails to upgrade in tandem with the changing knowledge level and functional needs of individuals, its utilization rate and effectiveness will tend to decline.

The health literacy and self-management ability of newly diagnosed T2DM patients show a continuous upward trend. This study demonstrated that within 6 months of diagnosis, patients with newly diagnosed T2DM exhibited a significant and sustained increase in health literacy and self-management skills (*P* < .001), which were mutually reinforcing. This trend is closely linked to the recent transformation in diabetes management practices. With the advancement of the “Healthy China 2030” initiative, the “first diagnosis diabetes education package” has been introduced in primary healthcare institutions. This initiative encourages patients to transition from passive treatment to proactive disease management and establishes an early intervention pathway where “diagnosis equates to intervention.” Systematic reviews indicate^[31]^ that disease education and lifestyle interventions at the time of initial diagnosis can markedly enhance health literacy. Early improvements in literacy, such as understanding the importance of blood glucose monitoring, can directly boost compliance with self-management practices like dietary control and regular medication. Self-management skills displayed a gradual improvement 6 months post-diagnosis, aligning with findings from related research both domestically and internationally.^[32,33]^ Simon qualitative study revealed^[34]^ that newly diagnosed patients experienced a significant increase in their perception of disease severity and susceptibility due to immediate health threats, such as uncontrolled blood glucose levels and the risk of acute complications. Consequently, they took proactive steps to manage their condition to mitigate these risks. This metabolic awareness effect motivated them to engage frequently with health education and actively learn about blood glucose monitoring and medication management, thereby laying the cognitive groundwork for enhanced self-management abilities. This echoes the conclusion emphasized by Babazadeh et al^[35]^ in their study on Pap smear screening behavior among married women, that health literacy is not only about information acquisition at the cognitive level, but also serves as an “action catalyst” for translating into actual health decisions and behaviors The newly diagnosed patients are highly sensitive to health information due to the “metabolic alertness” effect, which creates a golden window period for early health education intervention.

The relationship between chronic disease resource utilization, health literacy, and self-management behaviors of newly diagnosed T2DM patients. The cross-lag model was employed to uncover the staged interaction mechanism among chronic disease resource utilization, health literacy, and self-management behavior in newly diagnosed T2DM patients. During the initial phase of diagnosis (0–3 months), chronic disease resource utilization significantly and positively predicted health literacy (*β* = 0.24, *P* < .001). However, the reverse predictive effect of health literacy on resource utilization did not attain statistical significance (*β* = 0.08, *P* = .103). This 1-way relationship indicates that patients’ resource acquisition behavior in the early stage is significantly passively dependent,^[36]^ and their resource utilization primarily stems from the standardized supply of the medical system, such as initial diagnostic education courses and family doctor contracts, rather than active screening based on individual knowledge levels. Although this supply-driven resource utilization model can rapidly establish basic disease cognition, homogeneous intervention is prevalent in medical institutions,^[37]^ including the uniform distribution of standardized education manuals and group teaching. This may result in a mismatch between resource supply and individual patient demand. For instance, patients with lower education levels may experience a bottleneck in knowledge acquisition due to difficulties in understanding graphical data, while those with higher education levels may encounter information reception fatigue from repetitive content. Both scenarios can diminish the efficiency of resource conversion. The core contradiction at this stage is the tension between the medical system’s “supply-side standardization” and patients’ “demand-side heterogeneity,” suggesting that a baseline health literacy assessment should be incorporated into early interventions^[38]^ to facilitate the hierarchical classification of resource supply.

Six months post-diagnosis, the direct impact of resource utilization on self-management behavior diminished (*β* = 0.12, *P* = .087); yet, the indirect effect via health literacy remained significant (*β* = 0.067, *P* = .021). A chain transmission mechanism of “resource utilization, health literacy, and self-management behavior” was presented. This differs from Qian Yan cross-sectional study^[24]^ on the direct effect of “resource utilization–self-management behavior.” This shift reflects 2 key changes. On one hand, resource demand has become more refined and differentiated.^[39]^ Patients’ reliance on basic maintenance resources such as routine outpatient visits and blood glucose test strips has decreased, while their demand for specialized and personalized resources has significantly increased. Examples include guidance for insulin dose adjustment based on continuous glucose monitoring, customized nutrition programs according to the risk of complications, and real-time remote monitoring of exercise rehabilitation, among others. This escalation in demand and the improvement in health literacy create a 2-way mapping. When individuals can comprehend the complex relationship between blood glucose fluctuations and diet and exercise, they actively seek higher-order resource support, reflecting the cognitive behavior law of “ability improvement drives demand evolution.” On the other hand, the maintenance of self-management behaviors faces systemic challenges.^[40]^ With the increase in treatment complexity, such as insulin-intensive therapy dose adjustment and multi-drug combination management, and the weakened external supervision from the shift of high-frequency doctor-patient interaction from the first visit to routine follow-up, patients must rely on endogenous self-regulation mechanisms to sustain management behaviors. A cross-sectional study in China^[41]^ revealed that only 26.2% of newly diagnosed patients maintained stable self-management behaviors, indicating a significant behavior maintenance gap at this stage. Qualitative studies have shown that^[40]^ after achieving treatment goals such as short-term fasting blood glucose control, without progressive goal setting like long-term HbA1c management and complication prevention, it is easy for behavior to become slack. Additionally, social ecological changes, such as return-to-work time management conflicts and weakened family support, may also disrupt behavior persistence.

The indirect effect path of “resource utilization → health literacy → self-management behavior” discovered in this study aligns highly with the “knowledge → belief → behavior” transformation model of the American Diabetes Association,^[42]^ confirming the crucial role of health literacy as a mediating variable in the transformation of resource efficiency. This is highly consistent with the important mediating role mechanism of cognitive factors on behavior discovered in the research by Babazadeh et al.^[35]^ This implies that in clinical practice, a 3-dimensional assessment of “resources-literacy-behavior” should be introduced at the 6-month time point. It is essential not only to ensure the continuous availability of basic resources but also to enhance the efficiency of the transformation from literacy to behavior through scenario-based health guidance, such as training to respond to blood glucose fluctuations. Simultaneously, individualized resource supplementation should be provided based on specific needs, such as equipping elderly patients with intelligent medication reminder devices and developing fragmented exercise plans for working patients, to overcome the management dilemma of “diminishing marginal returns of resource supply.”

Based on the aforementioned findings and strengths, this study also identifies several limitations and points to directions for future research. First, limitation of sample representativeness: the sample in this study was drawn from a single medical center. Consequently, the results may be influenced by region-specific factors such as healthcare resource accessibility, local health policies, and cultural practices. Future research should conduct multicenter, cross-regional collaborative studies, incorporating broader and more diverse patient populations. This will help verify the generalizability of the findings and explore the adaptive adjustment of intervention strategies across different sociocultural contexts. Second, limitation of observation period: while the 6-month follow-up period effectively captures the formation patterns of early-stage behaviors, it is insufficient for evaluating the long-term maintenance of self-management behaviors, the stability of glycemic control, and the risk of long-term complications. Extending the observation period to 1 to 2 years or longer would help uncover the complete trajectory from “behavior establishment” to “outcome consolidation” and ultimately to “health outcomes,” thereby assessing the long-term benefits of early interventions. Third, insufficient systematic consideration of influencing factors: beyond the variables focused on in this study, self-management behaviors are profoundly influenced by multidimensional socio-ecological factors. Future research should integrate a richer set of variables, such as the quality of healthcare services, doctor-patient communication patterns, specific dimensions of family support (emotional, instrumental, informational), digital health literacy, and psychological resilience. More complex models, such as latent growth modeling or multilevel modeling, could be employed to construct a more comprehensive predictive and explanatory framework.

## 5. Conclusion

This study, which longitudinally tracked newly diagnosed T2DM patients, discovered that health literacy and self-management behaviors were mutually predictive during both the 0 to 3 months and 3 to 6 months post-diagnosis periods. The positive influence of chronic disease resource utilization on health literacy was primarily observed in the baseline period (0–3 months), and it indirectly improved self-management behaviors via health literacy. However, this effect diminished in the later stages. The study’s limitations include: the sample was sourced from a single region, which may have been affected by external factors such as regional medical resource allocation and cultural habits; the tracking period was limited to 6 months, making it challenging to observe the continuous evolution of individual behaviors and the impact of complications on self-management in long-term disease management. Future research could expand to include a multicenter sample, extend the observation period beyond 1 year, and comprehensively account for various socio-ecological factors to offer more comprehensive evidence to support the development of a precise intervention system.

## Author contributions

**Conceptualization:** Ming Fan, Weipeng Qian.

**Data curation:** Ming Fan, Weipeng Qian.

**Formal analysis:** Weipeng Qian.

**Funding acquisition:** Ming Fan.

**Writing – original draft:** Ming Fan, Weipeng Qian.
